# Adherence rates and risk factors for suboptimal adherence to secondary prophylaxis for rheumatic fever

**DOI:** 10.1111/jpc.15239

**Published:** 2020-12-19

**Authors:** Priya M Kevat, Ronny Gunnarsson, Benjamin M Reeves, Alan R Ruben

**Affiliations:** ^1^ College of Medicine and Dentistry James Cook University Cairns Queensland Australia; ^2^ Clinical Services Apunipima Cape York Health Council Cairns Queensland Australia; ^3^ Department of Paediatrics Cairns and Hinterland Hospital and Health Service Cairns Queensland Australia; ^4^ The Royal Children's Hospital Melbourne Victoria Australia; ^5^ Research, Development, Education and Innovation Primary Health Care Gothenburg Region Västra Götaland Sweden; ^6^ General Practice/Family Medicine, Primary Health Care, School of Public Health and Community Medicine, Institute of Medicine, Sahlgrenska Academy, University of Gothenburg Gothenburg Sweden; ^7^ Medical Services, Torres and Cape Hospital and Health Service Cairns Queensland Australia

**Keywords:** acute rheumatic fever, adherence, benzathine penicillin, concordance, rheumatic heart disease, secondary prophylaxis

## Abstract

**Aim:**

Secondary prophylaxis with 3–4 weekly benzathine penicillin G injections is necessary to prevent disease morbidity and cardiac mortality in patients with acute rheumatic fever (ARF) and rheumatic heart disease (RHD). This study aimed to determine secondary prophylaxis adherence rates in the Far North Queensland paediatric population and to identify factors contributing to suboptimal adherence.

**Methods:**

A retrospective analysis of data recorded in the online RHD register for Queensland, Australia, was performed for a 10‐year study period. The proportion of benzathine penicillin G injections delivered within intervals of ≤28 days and ≤35 days was measured. A multi‐level mixed model logistic regression assessed the influence of age, gender, ethnicity, suburb, Accessibility and Remoteness Index of Australia class, number of people per dwelling, Index of Relative Socio‐economic Advantage and Disadvantage, Index of Education and Occupation, year of inclusion on an ARF/RHD register and individual effect.

**Results:**

The study included 277 children and analysis of 7374 injections. No children received ≥80% of recommended injections within a 28‐day interval. Four percent received ≥50% of injections within ≤28 days and 46% received ≥50% of injections at an extended interval of ≤35 days. Increasing age was associated with reduced delivery of injections within 35 days. Increasing year of inclusion was associated with improved delivery within 28 days. The random effect of individual patients was significantly associated with adherence.

**Conclusions:**

Improved timely delivery of secondary prophylaxis for ARF and RHD is needed as current adherence is very low. Interventions should focus on factors specific to each individual child or family unit.

## What is already known on this topic

1. Acute rheumatic fever and rheumatic heart disease are prevalent among Australian Aboriginal and Torres Strait Islander paediatric populations, including in Far North Queensland.
2. Secondary prophylaxis with regular benzathine penicillin G injections is recommended to prevent disease morbidity and mortality.

## What this paper adds

1. Adherence to timely secondary prophylaxis for acute rheumatic fever and rheumatic heart disease in the Far North Queensland paediatric population is very low.
2. The most important factor for adherence was the ‘individual’ patient or family unit.
3. Improved delivery of secondary prophylaxis is essential. Interventions should be tailored to the individual child or family unit.

Acute rheumatic fever (ARF) and rheumatic heart disease (RHD) are prevalent in low‐income countries and certain disadvantaged populations in high‐income countries, including Australian Aboriginal and Torres Strait Islander children.^1^ The incidence of ARF among 5–14‐year‐old Aboriginal and Torres Strait Islander children was estimated to be 195 per 100 000 in 2013–2017, one of the highest known rates world‐wide.^2^ Disease burden is significant, with adverse outcomes of RHD, including heart failure, atrial fibrillation, endocarditis, stroke and death.^3^

Secondary prophylaxis with 3–4 weekly benzathine penicillin G (BPG) injections is necessary to prevent streptococcal infections and recurrent episodes of ARF that cause worsening RHD.^4^ BPG injections should be continued for a minimum of 10 years after the last episode of ARF or until the age of 21 (whichever is longer) if mild RHD is present, and until the age of 35 or 40 years minimum for moderate and severe RHD, respectively.^3^ The main challenge in secondary prophylaxis is low uptake of recommendations, with adherence rates below target threshold reported in a number of countries globally.^5^

In Australia, there have been major efforts to improve the detection and management of ARF and RHD. In 2009, the Rheumatic Fever Strategy saw the inception of a national coordination unit, RHD Australia, and support for register‐based state control programmes. Additionally, the End Rheumatic Heart Disease Centre of Research Excellence was established in 2015 to facilitate an evidence‐based approach to reducing disease. Despite programme strategies, adherence to secondary prophylaxis has been reportedly suboptimal in the Northern Territory, Western Australia, South Australia and Queensland.^6^, ^7^, ^8^, ^9^, ^10^, ^11^, ^12^, ^13^, ^14^, ^15^

Although de Dassel *et al*. found the Northern Territory register to underestimate adherence, it remained lower than recommended, with no significant effect on the key performance indicator of ≥80% adherence.^15^ In another study, de Dassel *et al*. demonstrated that receiving <80% of injections was associated with a four‐fold increase in the odds of ARF recurrence.^16^ Meanwhile Ralph *et al*. conducted a randomised clinical trial with multi‐component intervention to increase BPG prophylaxis delivery in the Northern Territory with no improvement.^12^ This suggests complex adherence and programme implementation challenges.

This study sought to determine rates of adherence to secondary prophylaxis in the paediatric population of Far North Queensland, Australia, where ARF and RHD are highly prevalent, and to explore factors associated with suboptimal adherence to secondary prophylaxis in order to guide programme strategies.

## Methods

### Ethics

Ethics approval for this study (reference number HREC/13/QCH/135‐881) was obtained from the Far North Queensland Human Research Ethics Committee.

### Data retrieval

Data from the period February 2004 to February 2014 were retrieved from Ferret, the official online database for registration of cases of ARF and RHD in Queensland. For each case matched to a unique identifier, the database custodian provided the age, date of birth, gender, ethnicity, suburb, RHD activity status, care plan/disease severity information and dates of BPG injections received. Accessibility and Remoteness Index of Australia (ARIA) classes for the suburbs were available from the Australian Population and Migration Research website. The suburbs were also matched to a Local Government Area and using publicly available census data (2011), the average number of people per dwelling in these was calculated. Local Government Area percentile rankings within Australia for two Socio‐economic Indexes measured by the Australian Bureau of Statistics from census data (2011) were also added to the data set. These were the Index of Relative Socio‐economic Advantage and Disadvantage and the Index of Education and Occupation.

### Eligibility criteria

Data for individuals aged 0–18 years residing in Far North Queensland who had an ‘active’ disease status and who received ≥5 injections in total were included in analysis. Those who received <5 injections in total were excluded as they were not considered to be established on a secondary prophylaxis programme, potentially due to revised diagnosis or incorrect registration on the Ferret database. Injection intervals that were between 20 and 365 days were included in analysis. Those outside of this range were considered to have a high likelihood of being unreliable due to inaccurate recording or medically recommended cessation of injections.

### Data analysis

The total number of injections and proportion of children who had ≥10%, ≥25%, ≥50%, ≥75%, ≥80% and 100% of their injections delivered at intervals of ≤28 and ≤35 days was calculated. Using case dates of birth and dates of injection delivery, the age at time of injection delivery was also calculated.

A multi‐level mixed model to explore potential risk factors for having injections delivered at intervals of >28 and >35 days, respectively, was developed using SPSS Statistics Software version 25, Chicago, Illinois. Independent fixed factors were age at injection, male gender, being of Aboriginal or Torres Strait Islander ethnicity, suburb, ARIA class, number of people per dwelling, Index of Relative Socio‐economic Advantage and Disadvantage, Index of Education and Occupation and calendar year of inclusion in the database. The individual person and suburb were both used as random effects. The level of significance was set to 0.05.

## Results

Two hundred and seventy‐seven children were included in analysis after eligibility criteria were applied, with a total of 7374 registrations of injections.

There were 146 males and 131 females (Table 1). Two hundred and fifty‐nine children were of Aboriginal and/or Torres Strait Islander background whilst the remaining 17 individuals were of another ethnicity. The mean age of children at the time of their inclusion in the database was 9.9 years (standard deviation 3.7) with an interquartile range (IQR) of 7.3–12 years. The majority of children lived in a location with an increased degree of remoteness demonstrated by ARIA score, and there was an average of 4.2 people per dwelling determined. Most patients lived in communities with a low Index of Relative Socio‐economic Advantage and Disadvantage and low Index of Education and Occupation.

**Table 1:** Demographic description of included children and injections delivered

	Age at diagnosis	Average number of people per dwelling	Index of Relative Socio‐economic Advantage and Disadvantage^†^	Index of Education and Occupation^‡^	ARIA score^§^	Number of injections delivered per individual
Number of individuals with data available	274	273	273	273	274	277
Mean	9.9	4.3	20	30	8.7	30
Median	9.9	4.4	6.0	15	12	27
Standard deviation	3.7	1.2	24	30	4.0	18
Interquartile range	7.3–12	2.9–4.9	4.0–27	3.0–65	4.2–12	14–41
Min	0.28	2.6	1.0	1.0	0.0	5
Max	18	6.6	91	90	12	94

^**†**^ A *low* percentile score indicates relatively greater disadvantage and a lack of advantage in general. For example, an area could have a low score if there are (among other things): many households with low incomes, or many people in unskilled occupations AND few households with high incomes, or few people in skilled occupations. A *high* percentile score indicates a relative lack of disadvantage and greater advantage in general. For example, an area may have a high score if there are (among other things): many households with high incomes, or many people in skilled occupations AND few households with low incomes, or few people in unskilled occupations.

^**‡**^ A *low* percentile score indicates relatively lower education and occupation status of people in the area in general. For example, an area could have a low score if there are: many people without qualifications, or many people in low skilled occupations or many people unemployed AND few people with a high level of qualifications or in highly skilled occupations. A *high* percentile score indicates relatively higher education and occupation status of people in the area in general. For example, an area could have a high score if there are: many people with higher education qualifications or many people in highly skilled occupations AND few people without qualifications or few people in low‐skilled occupations.

^§^ ARIA, Accessibility and Remoteness Index of Australia. Remoteness Classes: 1. Highly Accessible (ARIA score 0 to <0.20) – relatively unrestricted accessibility to a wide range of goods, services and opportunities for social interaction. 2. Accessible (ARIA score 0.20 to <2.40) – some restrictions to accessibility to some goods, services and opportunities for social interaction. 3. Moderately Accessible (ARIA score 2.40 to <5.95) – significantly restricted accessibility to goods, services and opportunities for social interaction. 4. Remote (ARIA score 5.95 to <10.5) – very restricted accessibility to goods, services and opportunities for social interaction. 5. Very Remote (ARIA score 10.5 to <15) – very little accessibility to goods, services and opportunities for social interaction.

In the included 7374 injections the mean age of children at the time of their injections was 12.5 years (standard deviation 3.35) with an IQR between 10.2 and 15.1 years. The mean number of injections delivered per individual was 30 (IQR 14–41).

Only 4% of children received at least half of their injections at intervals of ≤28 days (Table 2). Forty‐six percent of children received at least half of their injections within an extended 35‐day interval. No children received ≥80% of their injections within a 28‐day interval, which is a target recommendation.^3^ Twelve percent of children received ≥80% of their injections within 35 days.

**Table 2:** Injections given at intervals ≤28 days and ≤35 days (*n* = 277)

Proportion of injections given within investigated interval	Interval ≤28 days: Proportion of children % (*n*)	Interval ≤35 days: Proportion of children % (*n*)
≥10%	71% (198)	96% (266)
≥25%	22% (60)	84% (232)
≥50%	4.0% (11)	46% (128)
≥75%	0.36% (1)	15% (41)
**≥80%**	**0.0% (0)**	**12% (32)**
100%	0.0% (0)	0.72% (2)

Note: the significance of bold value is >= 80% of injections is a target recommendation for BPG delivery as per the Australian ARF/RHD guideline.

In the multi‐level regression model, it was found that the individual or family unit is significantly associated with adherence (Table 3). Older age at injection was associated with an increased risk for delivery of injections at longer intervals than 35 days with an odds ratio (OR) of 1.46 (1.24–1.71). Increasing year of inclusion was associated with reduced risk of delivery of injections at longer intervals than 28 days with an OR of 0.940 (0.896–0.987), but not for delivery of injections at longer intervals than 35 days. Gender, ethnicity, suburb, ARIA class, number of people per dwelling, Index of Relative Socio‐economic Advantage and Disadvantage and Index of Education and Occupation did not influence injection delivery.

**Table 3:** Risk factors for injection interval >28 days and >35 days (*n* = 7174)

Risk factor	Injection interval >28 days		Injection interval >35 days	
	*P* value	Odds ratio	*P* value	Odds ratio
Male gender	0.120	0.859 (0.708–1.04)	0.302	0.880 (0.691–1.12)
Aboriginal and/or Torres Strait Islander background	0.930	1.02 (0.678–1.53)	0.0664	1.64 (0.967–2.78)
Increased age at injection (5‐year intervals)	0.0802	0.841 (0.693–1.02)	0.000004	1.46 (1.24–1.71)
Increase by one person in the dwelling	0.863	1.01 (0.888–1.15)	0.478	1.07 (0.881–1.31)
Increase in Index of Relative Socio‐economic Advantage and Disadvantage	0.466	0.998 (0.991–1.00)	0.592	1.00 (0.993–1.01)
Increase in Index of Education and Occupation	0.198	0.997 (0.993–1.00)	0.281	1.00 (0.989–1.00)
Increasing age at diagnosis (5‐year intervals)	0.0826	1.22 (0.975–1.53	0.111	0.834 (0.667–1.04)
Increasing year of inclusion (year)	0.0121	0.940 (0.896–0.987)	0.920	0.997 (0.944–1.05)
Random effect: Individual	1.76 × 10^–8^ ^†^	—	7.77 × 10^–14^ ^‡^	—
Random effect: Suburb (includes ARIA)	‘Redundant’	—	0.289	—

^†^ Corrected Akaike information criterion: 34 181; Bayesian information criterion: 34 202.

^‡^ Corrected Akaike information criterion: 31 717; Bayesian information criterion: 31 737.

## Discussion

Overall, adherence to secondary prophylaxis for ARF/RHD in Far North Queensland over the study period was insufficient to provide prophylaxis against recurrences of ARF per current guidelines. The vast majority of injections were not delivered within the recommended 28‐day interval and a significant number were not even administered within 35 days. De Dassel *et al*. found that the risk of ARF recurrence did not decrease until ≈40% of doses had been administered and that receiving <80% of injections was associated with a fourfold increase in the odds of ARF recurrence. This supports an urgent need for improvement in injection delivery. The strongest identifiable contributing factor to variation of injections delivered was the individual effect, that is elements specific to the individual child or their family unit. This is the first study to statistically demonstrate this finding. Interpretation must reflect on social determinants of health and the socio‐political context in which health and illness frames are produced.^17^ The outcome of no improvement in BPG delivery in Ralph *et al*.'s clinical trial with multi‐component chronic care model intervention demonstrates the complexity of the challenges faced,^12^ and we postulate that an approach with strong individual focus may be more successful.

The adherence in this study was equivalent to or lower than adherence reported in other studies in the Northern Territory, Western Australia, South Australia and Queensland, which varied from 7% to 89%.^6^, ^7^, ^8^, ^9^, ^10^, ^11^, ^12^, ^13^, ^14^, ^15^ Low rates of adherence have also been reported in a number of studies globally.^5^ This study specifically addressed the recommended timeframe of each injection delivery of ≤28 days compared to other studies which, using a threshold of number of injections per calendar year or time period, may report higher adherence. It was noted that a greater number of injections had been delivered by 35 days. However, adherence at this time point was still suboptimal.

Increasing age was statistically significantly associated with injection delivery >35 days; however, there was a non‐significant trend towards reduced risk of injection delivery >28 days with increasing age (Table 3). We suggest that this may be because of two separate social groups of older children, those who attend boarding schools where rigorous adherence to 28‐day secondary prophylaxis regimens were enforced, and those not attending boarding schools who may be prone to >35‐day intervals for reasons such as increased mobility and greater bestowed responsibility for injections. Similarly, the more recent year of inclusion in the Ferret database was associated with reduced risk of injection delivery >28 days to a small degree (*P* < 0.05, OR 0.94). However, for injection intervals >35 days, there was no effect. This may reflect improved resources and initiatives to increase disease awareness, case registration and secondary prophylaxis delivery over time. We postulate that such initiatives may have been easier to implement in boarding schools enforcing the 28‐day regime. This theory is plausible although there is no hard data supporting it.

Gender did not influence adherence to secondary prophylaxis in this study. Engelman *et al*.,^18^ Musoke *et al*.^19^ and Ralph *et al*.^12^ also did not find an association between gender and adherence, and Stewart *et al*. found that men and women were equally likely to receive injections.^9^ Eissa *et al*., however, found that females were more likely to receive treatment.^6^

The Indexes of Relative Socio‐economic Advantage and Disadvantage and Education and Occupation were not found to be associated with adherence, though this was in the context of overall suboptimal adherence and lower socio‐economic status. Studies by Kumar *et al*.^20^ and Ralph *et al*.^12^ with a majority of participants having lower socio‐economic background also did not demonstrate a significant association between low socio‐economic background and non‐adherence.^20^ Existing literature presents conflicting results regarding the relationship between patients' parents' level of education and adherence to secondary prophylaxis. Bassili *et al*. described non‐adherence to be more common among children whose parents had lower levels of education and occupation,^21^ but Kumar *et al*. did not find an association between parents' level of education and patients' adherence to secondary prophylaxis.^20^

The number of people per dwelling in the household of a case was not predictive of better or worse adherence, which was also the case in Ralph *et al*.'s study.^12^ However, Gasse *et al*. found that a household with ≥6 people was protective against poor adherence, possibly due to older siblings being able to assist with health‐care seeking.^22^ In our study, the average number of people per dwelling was determined using census data and we found a mean of 4.3 people per dwelling (IQR 2.9–4.9). This may underestimate the true average number of people per dwelling for cases with ARF/RHD (and thereby underestimate an effect on adherence) as census data includes higher income households with a small number of people per dwelling and low likelihood of a residing case with ARF/RHD.

### Study limitations

The main limitation of this study is that the accuracy of the results is defined by the completeness of the database. Cross‐checking the Ferret database information with local registers and patient records was beyond what could be achieved within the resources available for this study. It is likely that there are individuals receiving injections that are not registered on the Ferret database and were therefore not included in our study; however, the extent is difficult to estimate. By comparison, an earlier study in a Northern Territory community in 2005 (prior to the Rheumatic Fever Strategy) found that the central register there contained 81% of patients identified in the community through other sources as eligible for inclusion.^6^ Being more recent, it is likely that this study had a similar or better coverage. De Dassel *et al*. found that the Northern Territory register underestimated mean adherence in the registered group by 3.8%; however, there was no significant effect on the key performance indicator of ≥80% adherence.^23^ Even if the Queensland register underestimates adherence to this degree, adherence remains substantially lower than current recommendations.

Aboriginal or Torres Strait Islander ethnicity and remoteness/accessibility were difficult to evaluate as factors given that a large proportion of patients were of Aboriginal or Torres Strait Islander background and were located in a place with some degree of remoteness. It may be that the effect of large distances of travel to secondary and tertiary centres and limited access to specialist services can be overcome by well‐functioning local primary care services and health‐care delivery.

An additional study limitation was that data pertaining to disease severity was not of sufficient reliability for analysis. There were many cases of duplicate care plans of differing severities and periods of time when individuals receiving injections were not registered for any care plan. In the opinion of two paediatricians working in the region, these care plans do not correlate well with actual clinical severity, especially as the latest classification of disease severity by echocardiogram was only established in 2012 and there was not a visiting paediatric cardiologist in the region able to verify disease severity until 2011.

### Study strengths

This study established rates of adherence to secondary prophylaxis for ARF and RHD in the Far North Queensland paediatric population over a 10‐year period. Analysis of adherence in this group over this length of time has not previously been described in the literature. To our knowledge, it is the first study in which a multi‐level model was used to evaluate the influence of factors including the effect of the individual, age, gender, Aboriginal or Torres Strait Islander status, suburb, ARIA class, number of people per dwelling, years since inclusion on a register and socio‐economic status on adherence to secondary prophylaxis for ARF and RHD.

## Conclusions

This study demonstrated suboptimal adherence to secondary prophylaxis for ARF and RHD in the Far North Queensland paediatric population and points to the importance of the unique child and their family unit in adherence to secondary prophylaxis. Interventions must identify particular individual or family unit factors of importance and endeavour to resolve barriers and promote enablers unique to the child and their family unit. Consideration of social determinants of health and reflection on health and illness discourses is integral to interpretation and formulation of joint solutions. A generic approach is highly unlikely to be successful.
